# Response to the Letter to the Editor

**DOI:** 10.1111/anec.12756

**Published:** 2020-03-10

**Authors:** Chaudry Nasir Majeed, Muhammad Imtiaz Ahmad, Irfan Ahsan, Muhammad Ali Anees, Sanjay K. Maheshwari, Elsayed Z. Soliman

**Affiliations:** ^1^ Department of Internal Medicine Section on Hospital Medicine Wake Forest School of Medicine Winston‐Salem North Carolina; ^2^ Department of Internal Medicine Section of Hospital Medicine Geisinger Medical Center Danville Pennsylvania; ^3^ Allama Iqbal Medical College Lahore Pakistan; ^4^ Liaquat University of Medical and Health Sciences Jamshoro Pakistan; ^5^ Department of Epidemiology and Prevention Epidemiological Cardiology Research Center (EPICARE) Wake Forest School of Medicine Winston‐Salem North Carolina; ^6^ Department of Internal Medicine Section on Cardiology Wake Forest School of Medicine Winston‐Salem North Carolina

We thank Hassing et al. for their interest in our publication (Majeed et al., 2019). In their letter to the editor, they expressed two concerns about the findings of our study. First, they thought that the QT interval was not heart rate adjusted in our study; however, this is not the case. We have corrected QT interval using NHANES specific QT correction formula as mentioned clearly in the method section of the manuscript (Soliman, Shah, Boerkircher, Li, & Rautaharju, 2014). This is a linear regression‐based method for QT correction. The American Heart Association (AHA), American College of Cardiology (ACC), and Heart Rhythm Society (HRS) for the Standardization and Interpretation of the ECG recommend using linear regression functions rather than the traditional Bazett's and Fridericia's formula (Rautaharju et al., 2009). Therefore, our observed association of total bilirubin with prolonged QT interval is not due to the association of bilirubin with heart rate, as suggested by Hassing et al.

Second, Hassing et al. pointed out a lack of adjustment for liver cirrhosis in our association of bilirubin with QT interval. We agree that liver cirrhosis has been associated with a prolonged QT interval (Tsiompanidis et al., 2018). Nevertheless, we adjusted our models for alanine aminotransferase and aspartate aminotransferase which can be elevated in liver disease especially in nonalcoholic fatty liver disease (Sehatpour et al., 2020), a common cause of liver cirrhosis in the United States (Negro, 2020). Also, our sample was from the general population and free of cardiovascular disease, and therefore, the likelihood of severe liver cirrhosis is unlikely. Finally, we adjusted for several potential confounders, and the observed association of bilirubin with a prolonged QT interval remained.

In their letter to the editor, Hassing et al. also report results of association of bilirubin with Fridericia's heart rate corrected QT interval in a sample of healthy participants aged 18–30 years. They observed a negative association of bilirubin with QT interval as opposed to the results of our study. Perhaps the authors should read the AHA/ACC/HRS recommendations for QT correction (Rautaharju et al., 2009) to realize that using nonlinear‐based correction such as Fridericia's is flawed, and that age, sex, and race differences in QT exist. In our study, we used linear‐based QT correction, and our study population cohort is represented by multiracial participants aged 40–90 years, not to mention the large sample. Therefore, it is odd to compare our more generalizable results to the results presented by Hassing et al.
